# Computed tomography radiomics to predict microsatellite instability status and immunotherapy response in gastric cancer

**DOI:** 10.1186/s13244-025-02050-1

**Published:** 2025-08-14

**Authors:** Zhou Li, Zixuan Ding, Yongping Lian, Yongqing Liu, Lei Wang, Pengbo Hu, Fangyuan Zhang, Yan Luo, Hong Qiu

**Affiliations:** 1 Department of Oncology, Tongji Hospital, Tongji Medical College, Huazhong University of Science and Technology, Wuhan, China; 2 Lanzhou University School of Medicine, Lanzhou, China; 3 Xiangyang Central Hospital, Xiangyang, China; 4 Department of Radiology, Tongji Hospital, Tongji Medical College, Huazhong University of Science and Technology, Wuhan, China

**Keywords:** Radiomics, MSI, Immunotherapy, Gastric cancer, Prognostic biomarkers

## Abstract

**Objectives:**

To develop and validate a CT radiomics model for predicting microsatellite instability (MSI) status in preoperative gastric cancer (GC) patients and to explore the underlying immune infiltration pattern of the radiomics model.

**Materials and methods:**

This study used three retrospective datasets from Tongji Hospital (*n* = 304, training set), Xiangyang Central Hospital (*n* = 48, external testing set 1) and public datasets from The Cancer Imaging Archive (TCIA) (*n* = 43, external testing set 2). The preoperative contrast-enhanced CT images of GC were evaluated. Radiomics features were extracted and selected to construct the radiomics model in the training set, and further validated in the other two external testing sets. The outcome cohort, including 68 advanced unresectable GC patients receiving immunotherapy, was used to assess the predictive value of the radiomics model for treatment response and outcomes. We analyzed RNA-sequencing data from TCIA to investigate the underlying genomics characterization and immune infiltration spectrum of the radiomics model.

**Results:**

Four radiomic features were ultimately selected to develop the radiomics model. The model demonstrated good predictive performance for MSI status, achieving AUCs of 0.952, 0.835, and 0.879 in the training set and the two external testing sets, respectively. Radiomics scores (Radscores) was an independent predictor for PFS in the outcome cohort (HR: 0.145; 95% CI: 0.032–0.657; *p* = 0.012). Radscores were positively correlated with CD8+ T cells (R = 0.74, *p* = 0.013) and negatively related to M2-type macrophages (R = −0.67, *p* = 0.028).

**Conclusion:**

Our CT radiomics model could effectively predict MSI status and immunotherapy outcomes in GC patients therefore, may act as a potential noninvasive tool for personalized treatment decisions.

**Critical relevance statement:**

Our study develops a noninvasive biomarker based on readily available imaging to identify gastric cancer patients who may benefit from immunotherapy. It also reveals biological meanings of the radiomics biomarker, promoting further research into interpretability and clinical application of radiomics.

**Key Points:**

- A CT-based radiomics model was constructed to noninvasively predict gastric cancer (GC) microsatellite instability status.
- This immune-related radiomics model can effectively predict immunotherapy outcomes in GC.
- This noninvasive method can serve as a supplement for treatment decisions.

**Graphical Abstract:**

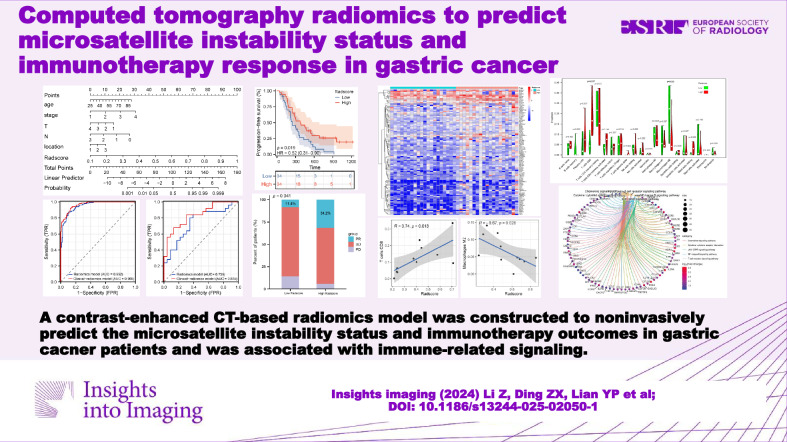

## Introduction

Gastric cancer (GC) is characterized by high heterogeneity and poor prognosis, and its drug treatment has always been a challenging focus in medical research [1, 2]. In recent years, with the advent of immunotherapy and targeted therapy, the management of gastric cancer has gradually become more precise [3]. Microsatellite instability (MSI) status is an emerging biomarker for predicting responses to immunotherapy. Microsatellite instability-high (MSI-H) gastric cancer has traditionally been considered a favorable population for immunotherapy [4], due to the high level of immune cell infiltration in MSI-H tumors [5]. Data from multiple clinical trials have confirmed the high response rate to immunotherapy in MSI-H GC patients [6]. The United States Food and Drug Administration (FDA) has approved pembrolizumab, an anti-programmed death-1 (PD-1) antibody, for treating solid tumors with MSI-H, including GC [7]. The National Comprehensive Cancer Network (NCCN) Guidelines recommended testing for MSI status in all newly diagnosed GC patients [8]. Therefore, the diagnosis of MSI status is critical for clinical guidance of immunotherapy in GC patients.

Currently, MSI status is primarily assessed by immunohistochemistry (IHC) and next-generation sequencing (NGS) on specimens obtained by surgical resection or gastroscopy biopsy [9]. However, information on MSI status acquired after surgery has a limited influence on the preoperative treatment schedule [10]. In addition, both biopsy and surgery are invasive procedures that may expose patients to surgery-related complications [11]. Thus, it is clearly valuable to develop a noninvasive method for accurately and dynamically assessing the MSI status. Radiomics represents a prospective noninvasive technique that extracts multidimensional quantitative features from medical imaging to explore the in-depth information on tumor biology [12, 13]. The predictive capability of imaging biomarkers in multiple cancer types has been demonstrated in many studies, especially in predicting responses to immunotherapy [14]. On this basis, researchers have verified the robust correlation between radiomics features and MSI status in colorectal cancer [15]. Although recently several studies have attempted to develop noninvasive CT-based radiomic models for predicting MSI status in GC [16, 17] with limited sample sizes, they failed to correlate their model with treatment response or patient survival, and lacked sufficient investigation of underlying genomic interpretations [16, 17].

This study aims to develop and validate a radiomics model based on pretreatment CT images for predicting the MSI status and immunotherapy responses in GC patients and to further explore the underlying genomics characterization and immune infiltrate spectrum of the radiomics model by analyzing the Shapley value and transcriptome sequencing data.

## Materials and methods

### Study patients

This retrospective study was approved by the institutional review board of Tongji Hospital of Tongji Medical College of Huazhong University of Science and Technology (approval number: TJ-IRB20201221) and Xiangyang Central Hospital (approval number: 2016L10518), and informed consent was waived.

**Fig. 1:**
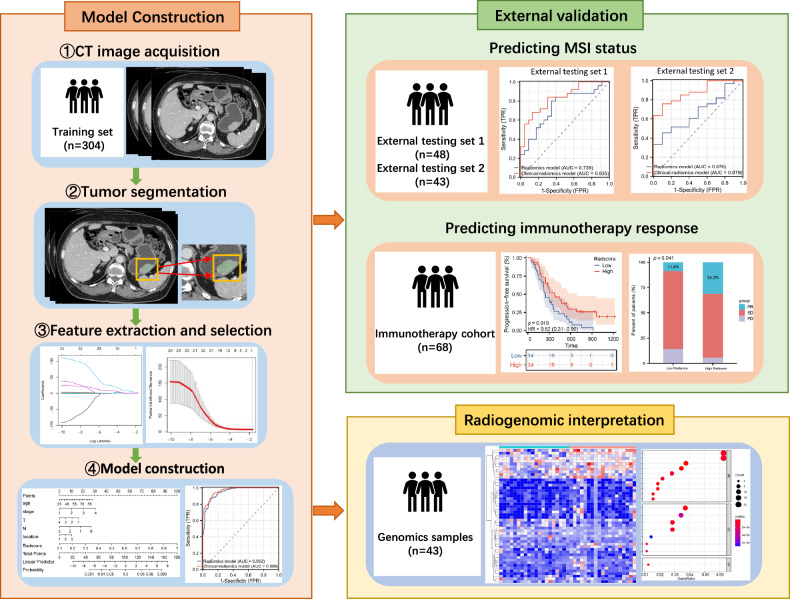
Study design for the construction and validation of a radiomics model to predict microsatellite instability status and immunotherapy response in gastric cancer (GC). The multicenter study framework comprises four distinct cohorts: (1) Training set (*n* = 304): retrospectively enrolled GC patients from Tongji Hospital of Tongji medical college of Huazhong University of Science and Technology; (2) External testing set 1 (*n* = 48): independent cohort from Xiangyang Central Hospital; (3) External testing set 2 (*n* = 43): Publicly accessible imaging and clinical data from The Cancer Genome Atlas (TCGA), with genomic profiling using the RNA-seq data from TCGA. (4) Immunotherapy cohort (*n* = 68): GC patients received immunotherapy at Tongji Hospital

To develop and externally validate the radiomic model, we retrospectively collected data of patients who received surgical resection for GC between January 2018 and December 2023 from two hospitals, including Tongji Hospital and Xiangyang Central Hospital. Inclusion and exclusion criteria were provided in the supplementary material. Eligible GC patients were also selected from The Cancer Imaging Archive and The Cancer Genome Atlas (TCIA/TCGA) databases. In this research, the data from Tongji Hospital were used as the training set to build the radiomics model, while data from Xiangyang Central Hospital and TCGA/TCIA databases were used as external testing sets (study design was shown in Figs. 1 and S1). Data from TCGA/TCIA databases, which included transcriptome sequencing information, were further used to explore the underlying genomics characterization and immune infiltration mechanism of our radiomics model. This study followed the standardized radiomics analyses protocols of METRICS [18] and CLEAR (Checklist for Evaluation of Radiomics research) [19]. Detailed documents of METRICS scores have been provided in the supplementary materials.

To evaluate the prognostic value of the radiomics model, we retrospectively included another outcome cohort from Tongji Hospital. Patients with unresectable advanced GC who received at least two cycles of immunotherapy as first-line treatment between January 2018 and December 2023, with available baseline CT and clinical follow-up data, were included.

### CT image acquisition

Abdominal contrast-enhanced CT scans were performed within 2 weeks before surgery using one of the following scanners: LightSpeed VCT, Optima CT660/680, Discovery CT750HD, Revolution (GE Healthcare), SOMATOM Force (Siemens Healthineers), Aquilion ONE CT (Toshiba Medical Systems Corporation) and Brilliance iCT256 (Philips). Specific parameters are detailed in the supplementary material.

### Tumor segmentation, radiomics features extraction and selection

Tumor segmentation was performed by an experienced researcher with 3 years of expertise in abdominal imaging who was unaware of the clinicopathological information by using 3D slicer software (version 5.0.3) on the portal venous phase images, where the whole tumor was manually outlined on each section and fused to create volumes of interest (VOIs). All segmentations were further confirmed by a senior radiologist specializing in abdominal imaging for 10 years, and disagreements were resolved by consensus.

Radiomics features extraction and selection were performed using an in-house software (MItalytics postprocessing software (FISCA Healthcare)). All VOIs were isotropic resampled to 1 × 1 × 1 mm voxel size. The Hounsfield units were resampled to 64 bins.

First, we performed a consistency test and calculated the intraclass correlation coefficient (ICC). Radiomics features with good intra- and inter-observer repeatability (ICC > 0.75) were chosen. Subsequently, univariate via Chi-square test was used to further identify features that were significantly correlated with MSI status. Finally, the final optimal radiomics features strongly associated with MSI-H were obtained by applying the ten-fold cross-validation method and the least absolute shrinkage and selection operator regression (LASSO) method.

### Model construction and validation

The features identified by LASSO analysis were incorporated into four different classifiers, including Naive Bayes, Logistic Regression, Support Vector Machine, and Random Forest, to construct four different models. During the training phase, repeated ten-fold cross-validation was applied to optimize feature selection and parameter tuning. Among the four candidate models, the model demonstrating the highest area under the receiver operating characteristic curve (AUC) value was ultimately selected as the optimal classifier for subsequent Radiomics model construction. The posterior probability of each patient, which refers to the radiomics model’s estimated likelihood of a patient belonging to a predefined clinical category [20], was calculated and utilized as the Radiomics scores (Radscores). GC patients with higher Radscores were more likely to be MSI-H. Univariate logistic regression analysis was conducted to identify significant clinical predictors for MSI-H subtypes to develop clinical-radiomics model.

Predictive performance of the models was assessed by receiver operating curve (ROC). The sensitivity, specificity and overall accuracy were calculated. The calibration curves and decision curve analysis (DCA) were employed to evaluate clinical effectiveness of the models.

### Prognostic significance of Radscores

In the outcome cohort, patients were stratified into high- and low-Radscores groups according to the cut-off value determined by X-tile software. X-tile algorithmically identifies cut-offs by iteratively testing all possible divisions of the Radscore distribution against survival [21]. The selected cut-off corresponded to the point of maximal statistical association assessed via log-rank test for survival endpoints. Kaplan–Meier survival curves were generated and compared using the log-rank test. ROC analysis was applied to evaluate the accuracy of Radscores in predicting overall survival (OS) and progression-free survival (PFS).

### Radiotranscriptomic analyses

Patients in the TCIA/TCGA cohort were categorized into low- and high-Radscores groups based on the cut-off value defined by X-tile software. Differential expressed genes (DEGs) between the high- and low-Radscores patients were identified by Wilcox test. Then, gene ontology (GO) and Tokyo encyclopedia of genes and genomes (KEGG) analysis were performed to identify the underlying molecular pathways associated with the Radscores.

### Statistical methods

The Mann–Whitney U test and Student’s *t*-test were used to compare the continuous variables, while Chi-square test was used for comparison of categorical variables. R software (version 4.1.0) and SPSS software (version 21.0) were used for statistical analyses. A *p*-value less than 0.05 was considered statistically significant in all analyses.

## Results

### Data baseline characteristics

A total of 395 eligible GC patients were included in this study, including 304 patients from Tongji Hospital, which were used as the training set, 48 patients from Xiangyang Central Hospital and 43 patients from TCGA/TCIA datasets, which were used as external testing sets. Table 1 summarizes the clinicopathological features of patients in the training and external testing sets. Table 2 shows the distribution of patient characteristics in the training set based on their MSI status. In the training set, 151 patients had MSI-H GC, and these patients were more likely to be female, older in age, with earlier clinical stage, and tumor location closer to the lower part of the stomach (Table 2, all *p* < 0.05).

**Table 1:** Clinical and pathologic characters of patients included in this study

Characteristics		Number of patients (%)			*p*-value
		Training set	Testing set 1	Testing set 2	
		(*n* = 304)	(*n* = 48)	(*n* = 43)	
Age (years), mean ± SD		60 ± 10	64 ± 9	64 ± 9	0.25
Gender	Female	113 (37.17)	14 (29.17)	7 (16.28)	0.36
	Male	191 (62.83)	34 (70.83)	36 (83.72)	
AJCC stage	I	49 (16.12)	1 (2.08)	1 (2.33)	0.002
	II	92 (30.26)	21 (43.75)	8 (18.60)	
	III	143 (47.04)	16 (33.33)	30 (69.77)	
	IV	20 (6.58)	10 (20.83)	4 (9.30)	
T	T1	33 (10.86)	1 (2.08)	0 (0)	0.12
	T2	40 (13.16)	4 (8.33)	1 (2.33)	
	T3	139 (45.72)	23 (47.92)	22 (51.16)	
	T4	92 (30.26)	20 (41.67)	20 (46.51)	
N	N0	105 (34.54)	14 (29.17)	10 (23.26)	0.46
	N1-3	199 (65.46)	34 (70.83)	33 (76.74)	
M	M0	284 (93.42)	38 (79.17)	41 (95.35)	0.001
	M1	20 (13.82)	10 (20.83)	2 (4.65)	
Location	Cardio or fundus	48 (15.79)	16 (33.33)	18 (41.86)	0.15
	Body	120 (39.47)	10 (20.83)	13 (30.23)	
	Antrum	136 (44.74)	22 (45.84)	12 (27.91)	
MSI status	MSI-H	151 (49.67)	23 (47.92)	10 (23.26)	0.82
	MSS	153 (50.33)	25 (52.08)	33 (76.74)	

*SD* standard deviation, *AJCC* American Joint Committee on Cancer

**Table 2:** Patient characteristics in the training set according to the MSI/MSS status

Characteristics		MSI-H	MSS	*p*-value
		(*n* = 151)	(*n* = 153)	
Age (years), mean ± SD		64 ± 10	60 ± 11	< 0.001
Gender	Female	69 (45.69)	44 (28.76)	0.002
	Male	82 (54.31)	109 (71.24)	
AJCC stage	I	37 (24.50)	12 (7.95)	< 0.001
	II	55 (36.43)	37 (24.18)	
	III	50 (33.11)	93 (60.78)	
	IV	9 (5.96)	11 (7.18)	
T	T1	24 (15.89)	9 (5.88)	< 0.001
	T2	26 (17.22)	14 (9.15)	
	T3	70 (46.36)	69 (45.10)	
	T4	31 (20.53)	61 (39.87)	
N	N0	74 (49.00)	31 (20.26)	< 0.001
	N1-3	77 (51.00)	122 (79.74)	
M	M0	142 (94.04)	142 (92.81)	0.666
	M1	9 (5.96)	11 (7.19)	
Location	Cardio or fundus	14 (9.27)	34 (22.22)	< 0.001
	Body	50 (33.11)	70 (45.75)	
	Antrum	87 (57.62)	49 (32.03)	

*SD* standard deviation, *AJCC* American Joint Committee on Cancer

### Construction of the radiomics model and diagnostic efficacy assessment

For each VOI, 1313 radiomics features were initially extracted, and 1201 radiomic features with the ICC greater than 0.75 were retained after consistency testing. Subsequently, these 1201 features were subjected to univariate analysis via the Chi-square test, and 190 features were identified as significantly associated with the MSI-H and MSS groups. Finally, four features were identified by LASSO analysis, including original shape Maximum 2D Diameter Row, log sigma 3 mm 3D glszm GrayLevel Vaiance, wavelet LHH firstorder Mean and wavelet LLL firstorder 90Percentile. The four features were incorporated into four different classifiers, and the Random Forest classifier achieved the highest AUC value (0.95 vs 0.70–0.77 for others, Table S1). Consequently, the Random Forest model was selected to construct the Radiomics model, and Radcores were calculated for each patient. The Radscores were higher in the MSI-H group compared to those in the MSS group in the training set (mean: 0.68 ± 0.15 versus 0.32 ± 0.14; *p* < 0.001), the external testing set 1 (mean: 0.53 ± 0.15 versus 0.39 ± 0.16; *p* = 0.003) and the external testing set 2 (mean: 0.50 ± 0.16 versus 0.41 ± 0.17; *p* = 0.110).

**Fig. 2:**
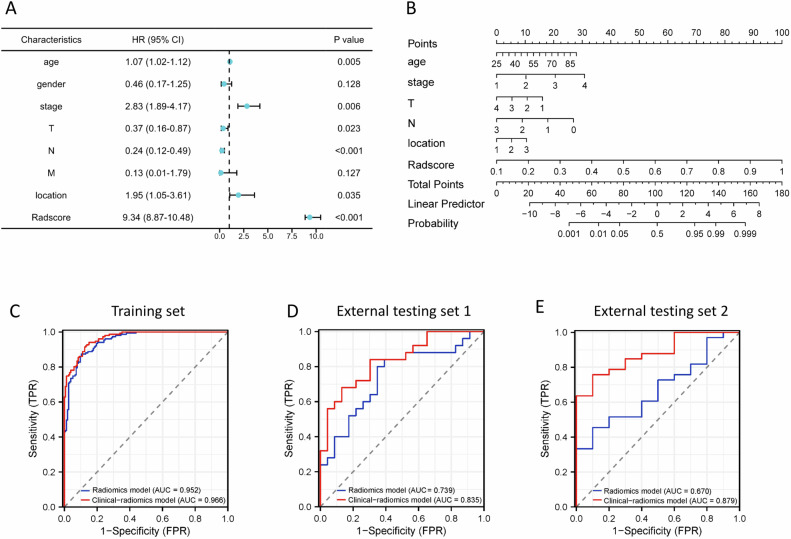
Construction and performance of the radiomics models for predicting MSI-H subtype. **A** Forest plot of predictors for MSI-H subtype in the training set. **B** The clinical-radiomics model presented with a nomogram scaled by the proportional regression coefficient of each predictor. **C**–**E** Performance of the radiomics model and the clinical-radiomics model for predicting MSI-H subtype with receiver operating characteristic curve analysis in the training set (**C**), external testing set 1 (**D**), and external testing set 2 (**E**)

Multivariate logistic regression analysis suggested that age, clinical stage, cT-stage, cN-stage, tumor location, and the Radscores were independent predictors for MSI-H subtype (Fig. 2A). A clinical-radiomics model integrating these six variables was developed and presented as a nomogram in Fig. 2B. The ROC analysis (Fig. 2C–E, Table 3) manifested that after incorporating clinical factors, the AUC of the clinical-radiomics model was superior to that of the radiomics model in the training set (0.966 [95% CI: 0.951, 0.982] vs 0.952 [95% CI: 0.931, 0.973]; *p* = 0.008), and the external testing set 2 (0.670 [95% CI: 0.493, 0.846] vs 0.879 [95% CI: 0.774, 0.984], *p* = 0.020), while there was no significant difference in AUCs between the clinical-radiomics model and the radiomics model in external testing set 1 (0.739 [95% CI: 0.596, 0.883] vs 0.835 [95% CI: 0.722, 0.947], *p* = 0.123). The DCA and calibration curves analysis of the models for the three datasets are shown in Fig. S2.

**Table 3:** Performance of the radiomics and clinical-radiomics models for predicting the MSI status

Dataset	AUC (95% CI)	Sensitivity	Specificity	Accuracy	PPV	NPV	Youden Index	*p*-value
Radiomics model								
Training set	0.952 (0.931–0.973)	0.874	0.895	0.885	0.892	0.878	0.770	-
Testing set 1	0.739 (0.596–0.883)	0.800	0.652	0.729	0.714	0.750	0.452	-
Testing set 2	0.670 (0.493–0.846)	0.900	0.455	0.558	0.333	0.938	0.355	-
Clinical-radiomics model								
Training set	0.966 (0.951–0.982)	0.940	0.850	0.895	0.861	0.935	0.790	0.0078
Testing set 1	0.835 (0.722–0.947)	0.680	0.870	0.770	0.850	0.714	0.550	0.1226
Testing set 2	0.879 (0.774–0.984)	0.900	0.758	0.791	0.529	0.962	0.658	0.0201

95% confidence intervals are presented in square brackets. *p*-value indicates the significance level of the comparison of AUCs with the radiomics model as the reference in the corresponding dataset

*AUC* area under the receiver operating characteristic curves, *CI* confidence interval, *PPV* positive predictive value, *NPV* negative predictive value

### Predictive value of the Radscores for immunotherapy outcomes

**Fig. 3:**
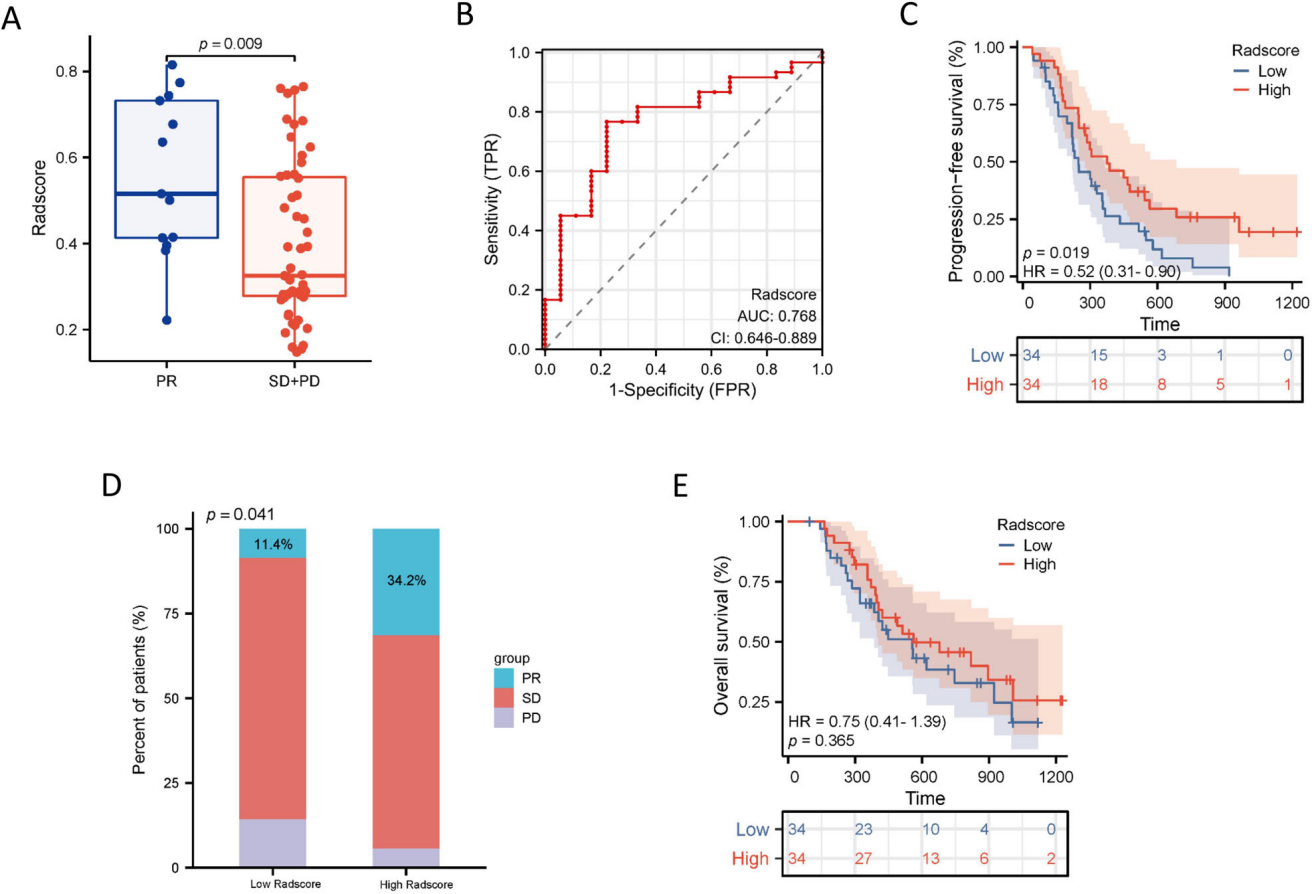
Performance of the radiomics model for treatment response prediction in the outcome cohort. **A** The relationship between the Radscores and the objective response to first-line immunotherapy. **B** Performance of the radiomics model for predicting PFS with ROC curve analysis in the outcomes cohort. **C** Kaplan–Meier analysis of PFS according to the Radscores (low or high, as defined by the X-tile). **D** Response rates in patients of the high- and low-Radscores groups. **E** Kaplan–Meier analysis of OS according to the Radscores (low or high, as defined by the X-tile)

Totally, 68 GC patients were enrolled in the outcome cohort. Baseline characteristics of patients were listed in Table S2. Treatment response was assessed with reference to the modified Response Evaluation Criteria in Solid Tumors (mRECIST), 12 (18%) patients had partial remission (PR), 48 (70%) with stable disease (SD), and 8 (12%) with progressed disease (PD). Patients in the PR group had higher baseline Radscores than patients in the SD and PD group (mean value: 0.56 ± 0.19 versus 0.38 ± 0.18; *p* = 0.009) (Fig. 3A).

At the end of the follow-up period, 43 patients were deceased, and 60 patients had progressive disease. For all patients, the median OS was 14.8 months (range from 3 to 41 months) and the median PFS was 9 months (range from 2 to 36 months). Multivariate COX regression analysis suggested high Radscores (HR: 0.145, 95% CI: 0.032, 0.657; *p* = 0.012) as an independent predictor of PFS after immunotherapy (Table 4). The AUC of the Radscores for predicting PFS was 0.768 in the outcome cohort (Fig. 3B).

**Table 4:** Uni- and multivariate Cox regression analysis for PFS in the outcome cohort

Variable	Univariable		Multivariable	
	HR (95% CI)	*p*-value	HR (95% CI)	*p*-value
Age				
(≤ 60 vs > 60)	1.001 (0.980–1.022)	0.938	1.005 (0.984–1.026)	0.631
Gender				
Female vs male	0.893 (0.530–1.503)	0.669	0.969 (0.564–1.664)	0.909
Clinical stage				
I vs II vs III vs IV	1.617 (1.036–2.525)	**0.034**	1.222 (0.665–2.248)	0.518
Radscores				
High vs low	0.156 (0.038–0.638)	**0.010**	0.145 (0.032–0.657)	**0.012**

*HR* hazard ratio, *CI* confidence interval

Bold values indicate statistically significant results where *p* < 0.05

Using the cut-off value of 0.384 determined by X-tile software, patients were stratified into high- and low-Radscores groups. The Kaplan–Meier curve shows that patients in the high-Radscores group showed significantly longer PFS than those in the low-Radscores group (*p* = 0.019, Fig. 3C). Besides, patients with high Radscores achieved a markedly higher partial remission rate (34.2%) than those with low Radscores (11.4%) (*p* = 0.041, Fig. 3D). However, the OS was not statistically significant between the high- and low-Radscore patients (*p* = 0.365, Fig. 3E).

### Gene expression differential analysis and functional enrichment analysis of the Radscores

**Fig. 4:**
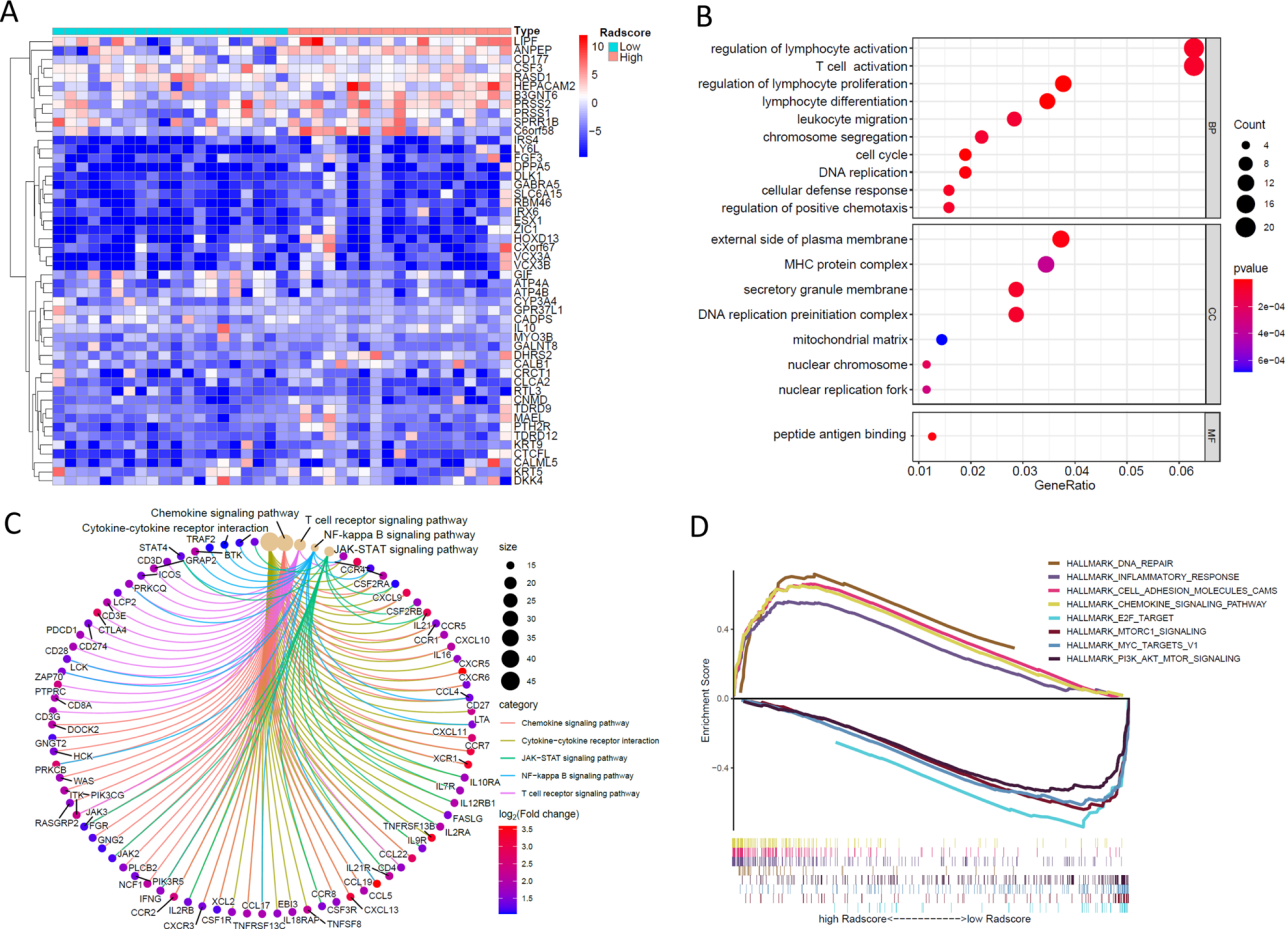
Molecular correlates of the radiomics model in gastric cancer. **A** Heatmap shows the expression levels of differentially expressed genes between groups stratified by the Radscores in the genomics sample. Row name of heatmap is the gene name, and the columns display sample groups with low-Radscores group on the left panel and high-Radscores group on the right panel. Each small square represents gene expression, with red representing genes with high expression, blue representing genes with low expression, and white representing genes with the same level of expression. **B** Bubble plots show the significantly correlated pathways with the Radscores based on the Gene Ontology (GO) functional enrichment analysis. The horizontal axis is the gene enrichment ratio, the vertical axis is the significant Biological Process (BP), Cell Components (CC), Molecular Functions (MF) terms. The size of the dots was determined by the number of enriched genes, and the color scale represents *p*-value. **C** Circos plots show the significantly correlated pathways with the Radscores based on the KEGG pathway enrichment analysis. The color and size of the nodes were determined by the enrichment degree value. Different colored lines represent significant category terms. **D** Enrichment scores of the representative pathways from the Hallmark gene sets database between the low- and the high-Radscores groups. Each line represents one particular gene set with unique color, and upregulated genes in high Radscore groups located in the left approaching the origin of the coordinates, by contrast the down-regulated lay on the right of *x*-axis

To explore the biological basics of the Radscores, the transcriptomic data and matched CT images of 43 GC patients from the TCGA/TCIA databases were utilized for the radiogenomics analysis. DEGs between high- and low-Radscore groups were shown in Fig. 4A. GO function and KEGG pathway analysis suggested that high Radscores were correlated with the upregulation of genes involved in the regulation of T-cell activation, cytokines interaction, and lymphocyte differentiation and proliferation (Fig. 4B, C). Gene set variant analysis indicated that the low-Radscore phenotype exhibited significant enrichment in hallmarks associated with aggressive tumors, such as MTORC signaling, MYC signaling, and PI3K-AKT-MTOR signaling pathway (Fig. 4D). Overall, these results were consistent with their unfavorable treatment response and prognosis.

### Immune cell infiltration pattern and immune enrichment analysis of the Radscores

**Fig. 5:**
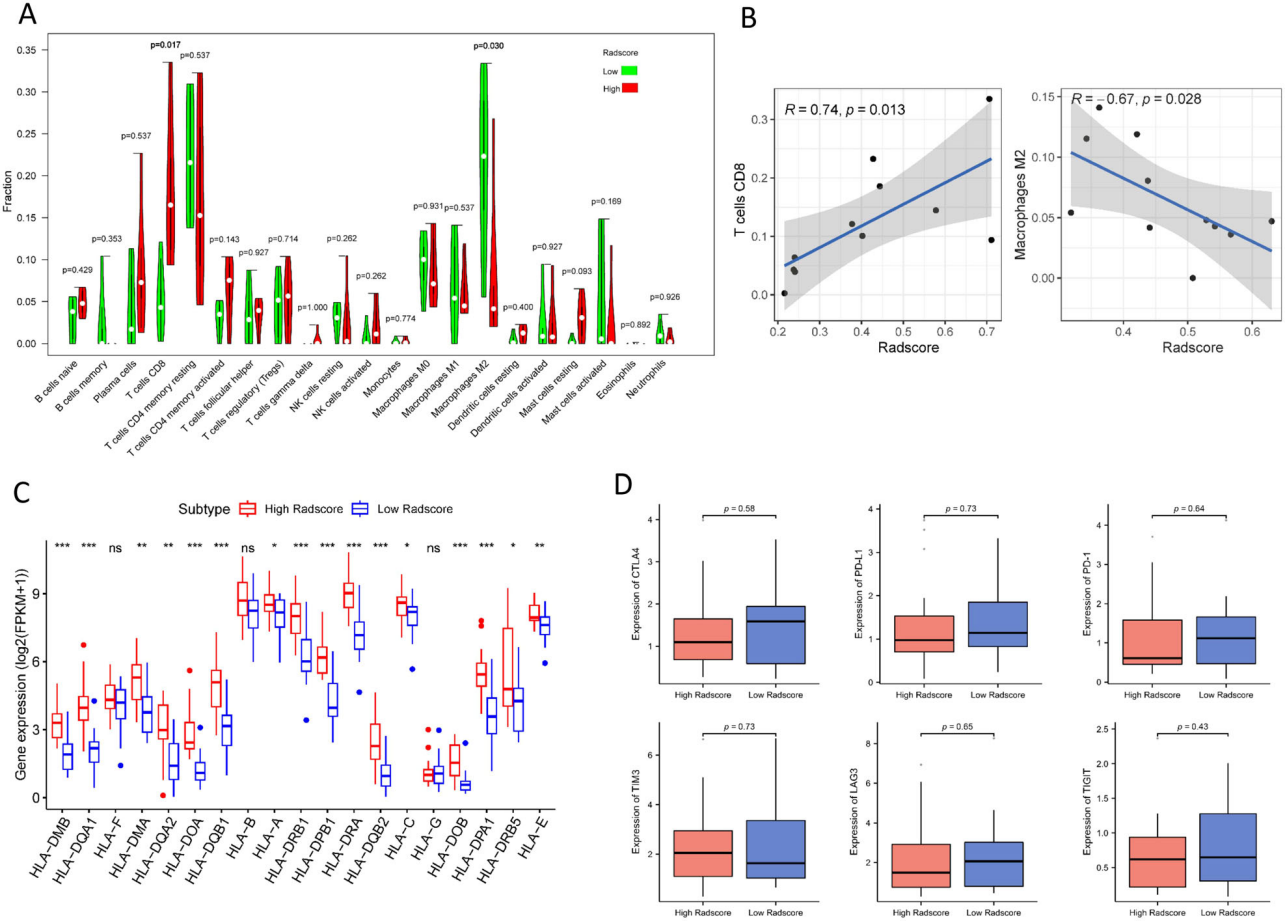
Radiotranscriptomic analysis of immune infiltration patterns associated with the radiomics model. **A** Vioplot shows the proportion of all kinds of immune cell subtype between groups stratified by the Radscores (low-Radscore: green color and high Radscore: red color) in the genomics sample. **B** Scatter plots show the relationship between the Radscores and CD8+ T cell and M2 macrophages. The blue line in each plot was fitted linear model indicating the proportion tropism of the immune cell along with Radscores. **C** The relationship between Radscores and the expression of HLA immune genes. Each column name corresponds to one HLA gene, and the *y*-axis indicates the normalized gene expression value. **D** The relationship between Radscores and immune checkpoints in GC. Each subplot represents a gene, with boxplots showing expression distributions for high-Radscore (red) and low-Radscore (blue) groups. The *y*-axis indicates normalized gene expression value, and *p*-values were calculated using the Mann–Whitney U test to assess significant differences between subgroups

Since the functional enrichment analysis described above showed that Radscores were correlated with immune-related signaling, we further performed immune cell infiltration and immune enrichment analysis on Radscores. The results indicated that patients with high-Radscores had a higher infiltration of CD8+ T cells compare to those with low-Radscores (*p* = 0.017), but the M2-type macrophage content was lower compared to those with low-Radscores (*p* = 0.030), (Fig. 5A). Meanwhile, correlation analysis showed that Radscores were positively correlated with CD8+ T cells (R = 0.74, *p* = 0.013) and negatively related to M2-type macrophages (R = −0.67, *p* = 0.028), (Fig. 5B). Moreover, a positive correlation was observed between Radscores and the expression of multiple HLA genes (Fig. 5C). Finally, we evaluated the relationship between Radscores and immune checkpoints including CTLA4, PD-1, PD-L1, TIM3, LAG3 and TIGIT. However, there were no significant associations between Radscores and these existing biomarkers of immunotherapeutic response (Fig. 5D).

## Discussion

In this study, a contrast-enhanced CT-based radiomics model was constructed to noninvasively predict the MSI status in patients with GC, with good performances in the training set and the two external testing sets. Moreover, our radiomics model was able to stratify GC patients to predict immunotherapy efficacy. Notably, our findings based on database RNA sequencing revealed the underlying molecular functional features and immune infiltrates spectrum (primarily immune-related signaling) related to our radiomics model.

Radiomics is increasingly acknowledged as a crucial virtual biopsy that can provide valuable data that may not be discernible through the unaided eye [22, 23]. Contrast-enhanced CT plays a key role in the clinical staging of GC, and is now a routine examination in preoperative GC patients [22, 24]. Numerous studies have emphasized the predictive utility of CT-based radiomics techniques in predicting preoperative pathology and survival in GC patients [25–27], because it is noninvasive and can be applied to patients unable to undergo surgery [28]. Although a previous study had proposed a similar predictive model based on MSI [29], our study included a larger number of cases, performed multi-step feature selection, and externally validated the robustness of the radiomics models through multicenter cohorts. Our radiomics model achieved fairly well predictive performance in the external testing dataset 1; however, the performance was suboptimal in the external testing dataset 2, which may be attributed to the heterogeneity in the CT acquisition, although in the data pre-processing stage, we resampled and standardized all the images to minimize the heterogeneity in different CT vendors. However, the use of positive enteric contrast material and other potential variations in CT acquisition parameters, as well as the small sample size, might also partly explain the low PPV (positive predictive value) in the external testing dataset 2. In any case, the radiomics model performed well in predicting MSI status, and the results were relatively stable and reproducible, suggesting that our method might be applicable to other GC patients. Furthermore, multivariate logistic regression analysis indicated that Radscores, age, clinical stage, and tumor location were independent predictors for the MSI status in GC, which is in accordance with previous reports [30, 31]. After combining Radscores with the above clinical factors that are readily accessible and noninvasive in routine clinical practice, a clinical-radiomics model was constructed and exhibited strong predictive performance.

Immunotherapy is one of the most important systemic treatments for GC, but not all patients respond to immunotherapy, and a validated marker of treatment response is limited [32]. In addition, the use of immunotherapy in non-responsive populations carries an increased risk of immune-related adverse events (irAEs) [33], so it is important to develop reliable predictive biomarkers for identifying advantaged populations that could benefit from immunotherapy. MSI status has traditionally been considered a biomarker for response to immunotherapy [34–36]. Although radiomic models based on MSI have been established in previous studies [28], few previous studies have validated the performance of the models in predicting immunotherapy response and prognosis in the follow-up cohort [37]. Our study demonstrated that a high Radscore (likely indicative of MSI-H states) was correlated with prolonged PFS in GC patients receiving immunotherapy, suggesting that our model is capable of predicting immunotherapy efficacy. We further revealed a potential link between CT imaging-derived Radscores, MSI status and clinical outcomes after immunotherapy, which may promote the application of radiomics-based identification of MSI status into clinical practice and has the potential to guide the selection of patients most likely to respond to immunotherapy. However, our radiomics model showed no significant contribution to risk stratification based on OS, possibly because of the limited number of patients in the outcome cohort and the fact that OS is related to many other factors, such as age, comorbidities and other posterior line treatment options.

The exploration of the biological significance of radiomics remains a scientific challenge at present [31]. Few previous studies had developed and validated CT radiomics model with biological understanding for pretreatment prediction of MSI status and immunotherapy efficacy in GC [38]. Combining the available public database resources and the advantages of bioinformatics algorithms may disclose potential connections between radiomic phenotypes and the tumor biology [39]. The results of Radiotranscriptomic analyses suggested that our radiomics model was correlated with T-cell activation and chemokine regulation. Further correlation analyses showed that Radscores were mainly related to CD8+ T cells and M2-type macrophages. In the tumor microenvironment (TME), CD8+ T cells play a major role in killing tumor cells [40], while M2-type macrophages contribute to a suppressive immune microenvironment, with both types of cells influencing the response to immunotherapy [41–43]. These results partly explained the model’s prediction of immunotherapy efficacy from the perspective of immune infiltration and provide theoretical evidence for the model to predict therapeutic efficacy and guide personalized immunotherapy. In addition, the absence of correlation between the model and immune checkpoints, which are conventional markers, suggests that the radiomics model may be used as a new marker, independent or in conjunction with conventional markers, to guide immunotherapy.

However, this study faces several constraints. First, the radiomics model was developed from retrospective data, which may introduce selection bias. To mitigate this issue, we performed rigorous validation on independent datasets that included two external institutions and adjusted for clinicopathologic factors in the analysis. Second, the sample sizes of the datasets and cohort were limited, and future prospective research with larger samples is required to further investigate the generalizability of the models, and other modeling algorithms such as deep learning might be applied. Finally, larger samples are needed for radiotranscriptomic analyses to validate the findings of this study.

## Conclusion

We developed and externally validated a CT radiomics model for noninvasive assessment of MSI status in GC. The radiomics model can effectively predict response to immunotherapy and outcomes in GC, which may serve as a potential tool for individualized treatment decisions. Notably, the radiomics model mainly correlated with the regulation of immune-related signaling.

## Supplementary information

ELECTRONIC SUPPLEMENTARY MATERIAL

## Data Availability

Data will be made available on request.

## Abbreviations

DCA: Decision curve analysis
DEGs: Differential expressed genes
GC: Gastric cancer
GO: Gene ontology
ICC: Intraclass correlation coefficient
KEGG: Tokyo encyclopedia of genes and genomes
LASSO: Least absolute shrinkage and selection operator regression
MSI: Microsatellite instability
MSI-H: Microsatellite instability-high
MSS: Microsatellite stability
OS: Overall survival
PR: Partial remission
PD: Progressed disease
PD-1: Anti-programmed death-1
PFS: Progression-free survival
ROC: Receiver operating curve
SD: Stable disease
TCIA/TCGA: The Cancer Imaging Archive and The Cancer Genome Atlas
VOIs: Volumes of interest
