# Supplementary material for: Computed tomography radiomics to predict microsatellite instability status and immunotherapy response in gastric cancer
Source: Insights Imaging. 2025 Aug 14;16:177. doi: 10.1186/s13244-025-02050-1 (PMC12354409; doi:10.1186/s13244-025-02050-1)
Supplement: Supplementary file 1 — ELECTRONIC SUPPLEMENTARY MATERIAL [file 13244_2025_2050_MOESM1_ESM.pdf]

Computed Tomography Radiomics to Predict Microsatellite Instability Status and Immunotherapy Response in Gastric Cancer

ELECTRONIC SUPPLEMENTARY MATERIAL

Appendix S1

Methodological supplement:

Study patients

The following were the inclusion criteria: (1) Patients were pathologically confirmed with gastric adenocarcinoma; (2) No anti-tumor treatment was administered before surgery; (3) Preoperative abdominal contrast-enhanced CT examination was conducted within two weeks; and (4) The MSI status was determined by IHC analysis. Exclusion criteria were: (1) Poor quality of CT images or lesions were too small to detect or delineate; and (2) Patients received any anti-tumor treatment before surgery.

Since the reported incidence of MSI-H GC in all GC is approximately 9-22% [1], in order to avoid large difference in numbers between the MSI-H and microsatellite stability (MSS) cases, we included cases successively with a ratio of approximately 1:1, and the inclusion time of MSI-H cases was from January 2018 to December 2023, and MSS cases from April 2020 to July 2023.

Clinical information, including age, gender, clinical TNM staging, tumor grade, maximal tumor diameter, tumor site, pretreatment serum CEA, CA199 and CA724 level, and HER-2, P53, and Ki-67 status of tumor tissue were collected. The MSI status was independently determined by a gastrointestinal pathologist who was blinded to the clinical information by evaluating the expression of mismatch repair (MMR) proteins (MLH1, MSH6, PMS2, and MSH2) in IHC-stained pathological sections. The complete deletion of any of the four MMR proteins was defined as MSH-H, while the presence of all four MMR proteins was defined as MSS.

Tumor segmentation, radiomics features extraction and selection

To further evaluate the intra- and inter-observer reproducibility of radiomics features, 30 random cases were selected and re-segmented by the same researcher after a four-week interval and by another researcher specializing in abdominal imaging for six years.

Model Construction and Validation

For constructing the clinical-radiomic combined model, univariate logistic regression analysis was conducted to identify significant clinical predictors for MSI-H subtypes in the training set. The identified clinical variables were then integrated with the Radscores using multivariate logistic regression analysis to develop clinical-radiomics model.

Prognostic Significance of Radscores

Univariate and multivariate Cox regression analyses were performed to determine the prognostic value of the Radscores and clinical factors.

Radiotranscriptomic Analyses

The proportional composition of different immune cell subtypes within each sample was estimated by the CEBERSORT algorithm, and immune cell infiltration between different Radscores groups was evaluated. Moreover, associations between the Radscores and immune checkpoints and existing biomarkers of immunotherapy was assessed.

Specific radiomic parameters used in this study:

The following parameters were used: tube voltage, 100-120 kV; automatic tube current modulation, 200-350 mA; matrix, 512 × 512; scan slice thickness, 5 mm and reconstructed slice thickness, 1.25 mm.

Non-ionic contrast medium Iopromide (Ultravist 370 mg iodine /mL, Bayer Healthcare,), Iohexol (Omnipaque 300 mg iodine/mL, GE Healthcare) or

Iodixanol (Visipaque 320 mg iodine/mL, GE Healthcare) was injected intravenously at a rate of 3-4 ml/s followed by a 25 ml saline flush. Bolus tracking technique was used to automatically trigger arterial phase, portal venous phase and delayed phase acquisition 5-8 seconds, 20-25 seconds and 180-240 seconds after the attenuation of abdominal aorta reached 150 HU, respectively.

Table S1 The area under the receiver operating characteristic curve value of the four different classification models in the training set

**Classification models**	**AUC**	**95% CI**
Random Forest	0.952	0.931-0.973
Naive Bayes	0.773	0.722-0.825
Support Vector Machine	0.704	0.646-0.762
Logistic Regression	0.702	0.644-0.760

Note: AUC: area under the receiver operating characteristics curves.

Table S2 Baseline characteristics of patients in the outcome cohort

**Characteristcs**		**Number of patients**
**Age(y), mean±SD**		60±11
**Gender**	female	29 (42.65)
	male	39 (57.35)
**AJCC stage**	Ⅰ	1(1.47)
	Ⅱ	1(1.47)
	Ⅲ	30(44.12)
	Ⅳ	36(52.94)
**Radscore**	high	34 (50.00)
	low	34 (50.00)
**Location**	Cardio or fundus	14 (20.59)
	Body	29 (42.65)
	Antrum	25 (36.76)

Note: SD: standard deviation; AJCC: American Joint Committee on Cancer.

Figure S1 Patient recruitment process flowcharts. (A) Training set and two external testing set. (B) Outcome cohort. (C) Genomics sample. GC: gastric cancer, TCGA: The Cancer Genome Atlas, TCIA: The Cancer Imaging Archive. Institution 1 indicates The Tongji Hospital of Tongji medical college of Huazhong University of Science and Technology, Institution 2 indicates Xiangyang Central Hospital.

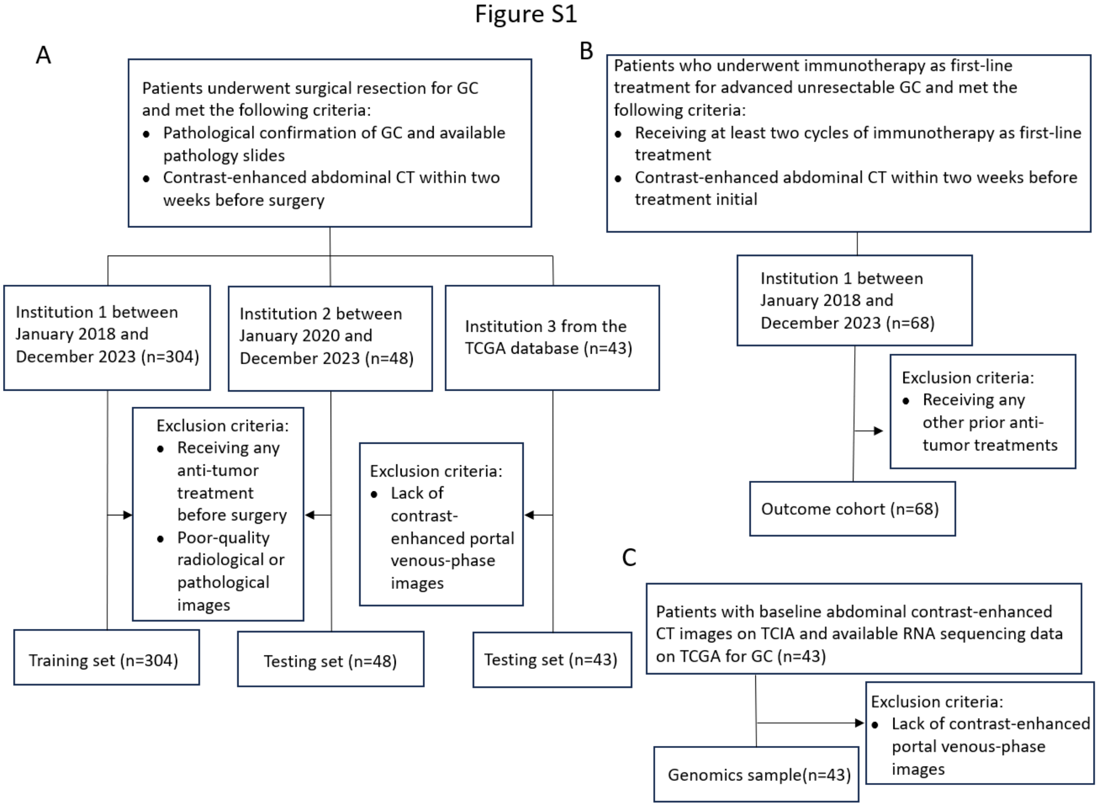

Figure S2 The DCA and calibration curves analysis of the models for the three datasets. The DCA and calibration curves of training set (A, D), external testing set 1 (B, E) and external testing set 2 (C, F).

Figure S2

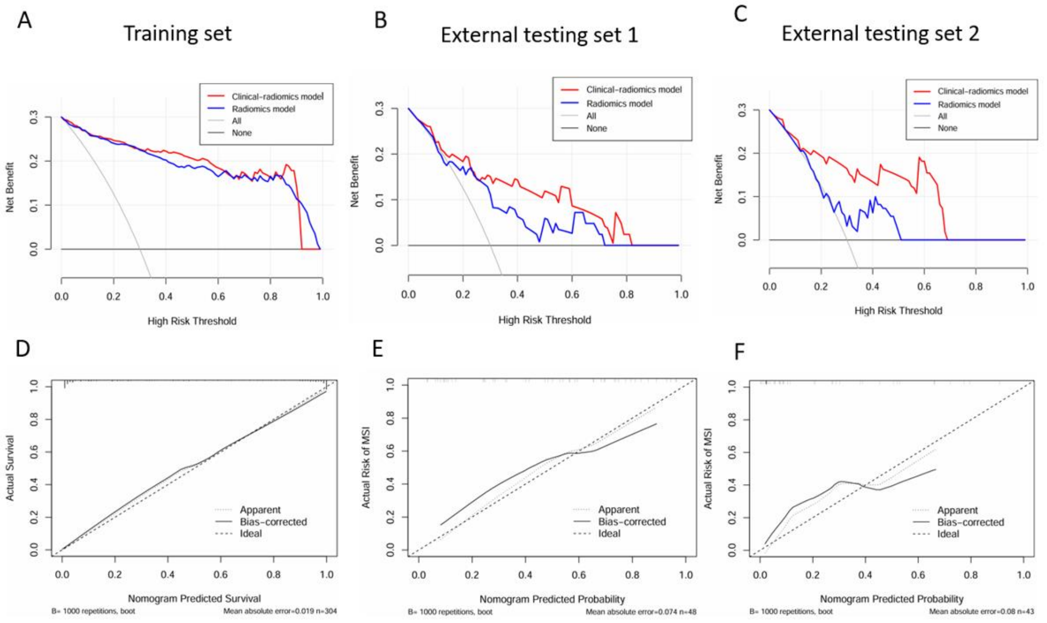

Reference:

1. Dudley JC, MT Lin, DT Le et al (2016) Microsatellite Instability as a Biomarker for PD-1 Blockade. Clin Cancer Res 22:813-20

METRICS Tool v1.0

Please fill out all conditions first for relevant sections and then all active items to calculate METRICS score.

Please note that default option is "No".

? Stands for explanation of items and conditions.

C Stands for conditional items or sections.

Items/Conditions	Definitions	Weights	Options
**Study Design**			
Item#1	? Adherence to radiomics and/or machine learning-specific checklists or guidelines	0.0368	◉ Yes ○ No
Item#2	? Eligibility criteria that describe a representative study population	0.0735	◉ Yes ○ No
Item#3	? High-quality reference standard with a clear definition	0.0919	◉ Yes ○ No
**Imaging Data**			
Item#4	? Multi-center	0.0438	◉ Yes ○ No
Item#5	? Clinical translatability of the imaging data source for radiomics analysis	0.0292	◉ Yes ○ No
Item#6	? Imaging protocol with acquisition parameters	0.0438	◉ Yes ○ No
Item#7	? The interval between imaging used and reference standard	0.0292	◉ Yes ○ No
**Segmentation** C			
Condition#1	? Does the study include segmentation?		◉ Yes ○ No
Condition#2	? Does the study include fully automated segmentation?		○ Yes ◉ No
Item#8	? Transparent description of segmentation methodology	0.0337	◉ Yes ○ No
Item#9	? Formal evaluation of fully automated segmentation C	0.0225	○ Yes ○ No
Item#10	? Test set segmentation masks produced by a single reader or automated tool	0.0112	○ Yes ◉ No
**Image Processing and Feature Extraction**			
Condition#3	? Does the study include hand-crafted feature extraction?		◉ Yes ○ No
Item#11	? Appropriate use of image preprocessing techniques with transparent description	0.0622	◉ Yes ○ No
Item#12	? Use of standardized feature extraction software C	0.0311	◉ Yes ○ No
Item#13	? Transparent reporting of feature extraction parameters, otherwise providing a default configuration statement	0.0415	◉ Yes ○ No
**Feature Processing**			
Condition#4	? Does the study include tabular data?		◉ Yes ○ No
Condition#5	? Does the study include end-to-end deep learning?		○ Yes ◉ No
Item#14	? Removal of non-robust features C	0.0200	◉ Yes ○ No
Item#15	? Removal of redundant features C	0.0200	◉ Yes ○ No
Item#16	? Appropriateness of dimensionality compared to data size C	0.0300	◉ Yes ○ No
Item#17	? Robustness assessment of end-to-end deep learning pipelines C	0.0200	○ Yes ○ No
**Preparation for Modeling**			
Item#18	? Proper data partitioning process	0.0599	◉ Yes ○ No
Item#19	? Handling of confounding factors	0.0300	◉ Yes ○ No
**Metrics and Comparison**			

Item#20	?	Use of appropriate performance evaluation metrics for task	0.0352	Yes	No
Item#21	?	Consideration of uncertainty	0.0234	Yes	No
Item#22	?	Calibration assessment	0.0176	Yes	No
Item#23	?	Use of uni-parametric imaging or proof of its inferiority	0.0117	Yes	No
Item#24	?	Comparison with a non-radiomic approach or proof of added clinical value	0.0293	Yes	No
Item#25	?	Comparison with simple or classical statistical models	0.0176	Yes	No
**Testing**					
Item#26	?	Internal testing	0.0375	◉ Yes	○ No
Item#27	?	External testing	0.0749	◉ Yes	○ No
**Open Science**					
Item#28	?	Data availability	0.0075	○ Yes	◉ No
Item#29	?	Code availability	0.0075	○ Yes	◉ No
Item#30	?	Model availability	0.0075	○ Yes	◉ No
			**Total METRICS score:**	**93.4%**	
			**? Quality category:**	**Excellent**	
			**? Publication ID:**	INSI-D-	

If you publish any work which uses this tool, please cite the following publication:

Kocak B, Akinci D'Antonoli T, Mercaldo N, et al. METhodological RadiomICs Score (METRICS): a quality scoring tool for radiomics research endorsed by EuSoMII. Insights Imaging. 2024;15(1):8. Published 2024 Jan 17. doi:10.1186/s13244-023-01572-w
